# Assessing the Safety and Aesthetic Benefits of Reduced Port Bikini-Line Sleeve Gastrectomy (RBSG): An Initial Report

**DOI:** 10.1007/s11695-025-08176-x

**Published:** 2025-08-20

**Authors:** Abdelkarem Ahmed Abdelkarem Mohamed, Mostafa Mahmoud Abdelfatah, Mohamed Nasr Shazly, Mina Adolf Helmy, Ahmed Nasr Shazly, Mohamed Saber Mostafa

**Affiliations:** 1 General Surgery Department - Kasralainy School of Medicine, Cairo University, Giza, Egypt; 2 Anesthesia Department, Armed Forces Hospital, Southern Region, Saudi Arabia; 3 Anesthesia Department, Ahmed Maher Teaching Hospital, Cairo, Egypt; 4 Anesthesia Department - Kasralainy School of Medicine, Cairo University, Cairo, Egypt

**Keywords:** Reduced port bikini-line sleeve gastrectomy, Body image, Port-site complications, Metabolic surgery, Laparoscopic sleeve gastrectomy

## Abstract

**Background:**

Obesity significantly increases the risk of chronic conditions and has profound psychological effects. While conventional laparoscopic sleeve gastrectomy (CLSG) effectively addresses these issues, concerns about visible scarring remain. Reduced port bikini-line sleeve gastrectomy (RBSG) was introduced to enhance cosmetic outcomes by repositioning ports along the bikini line, potentially improving body image satisfaction.

**Methods:**

This prospective cohort study included 118 patients who underwent RBSG at a single institution between September 2022 and September 2023. The study evaluated operative time, intraoperative complications, postoperative pain, port-site complications, and body image perception using the body image scale (BIS). Propensity score matching was applied to mitigate confounding variables, and statistical analyses included paired *t*-tests and non-parametric alternatives as appropriate.

**Results:**

The mean operative duration was 48.97 ± 8.51 min, with a 1.7% incidence of inferior epigastric artery injury, effectively managed intraoperatively. Port-site hernias and surgical site infections occurred in 1.7% and 3.4% of cases, respectively. BIS scores improved significantly from 18.58 ± 1.87 preoperatively to 11.44 ± 1.99 postoperatively (*p* < 0.001), indicating enhanced body image perception. Pain scores remained low at 6 and 12 h postoperatively, and no significant correlation was found between pain levels and port-site complications.

**Conclusions:**

RBSG is a safe and effective alternative to CLSG, offering equivalent weight loss and safety profiles while significantly improving postoperative body image perception. The technique maintains technical feasibility without compromising operative time or increasing complications. Larger, multicenter studies with extended follow-up are warranted to validate these findings and assess the long-term sustainability of the observed benefits.

**Supplementary Information:**

The online version contains supplementary material available at 10.1007/s11695-025-08176-x.

## Introduction

Obesity is a multifactorial disease characterized by excessive adiposity, which markedly increases the risk of developing numerous associated medical conditions, including type 2 diabetes mellitus, cardiovascular diseases, and certain malignancies [1–13]. Projections suggest that by 2035, over 50% of the global population may be classified as overweight or obese, with an alarming statistic indicating that 25% of individuals will meet the criteria for obesity [14, 15]. Beyond its physical health implications, obesity also exerts considerable psychological and social consequences, often resulting in diminished quality of life, stigmatization, and mental health disorders like depression and anxiety [16–18].

Metabolic and Bariatric Surgery (MBS) has been established as the most effective long-term intervention for morbid obesity, facilitating sustainable weight loss and significant metabolic enhancements. Among the various surgical options, laparoscopic sleeve gastrectomy (LSG) has emerged as the predominant metabolic procedure globally, owing to its favorable safety profile and technical straightforwardness relative to alternatives like Roux-en-Y gastric bypass and One-anastomosis Gastric Bypass [19, 20]. CLSG functionally reduces gastric capacity, leading to alterations in gut hormone secretion that promote weight loss and metabolic improvement [21–23].

Despite its efficacy, CLSG is not without drawbacks, particularly regarding cosmetic outcomes and patient satisfaction concerning surgical scars [18]. Although minimally invasive, the incisions can result in prominent postoperative scars, and studies indicate that a substantial portion of patients express dissatisfaction with their abdominal scars post-surgery [18, 24]. While CLSG significantly improves patients’ quality of life by addressing obesity-related conditions, visible scarring can contribute to body image concerns and emotional distress and may deter potential candidates from pursuing surgical options [18, 25, 26].

In response to the aesthetic shortcomings of the conventional laparoscopic port-site of sleeve gastrectomy (CLSG), several surgical modifications have been introduced, including single-incision Sleeve Gastrectomy (SISG) and reduced-port sleeve gastrectomy (RPSG) [27–29]. SISG is characterized by a single umbilical incision that enhances cosmetic outcomes but introduces significant technical challenges, such as diminished triangulation, instrument crowding, and restricted maneuverability—often necessitating specialized instruments for effective execution [28, 30]. Additionally, single-incision approaches are associated with increased risks of incisional hernia and postoperative pain due to the large umbilical port [30–32]. Similarly, RPSG, designed to reduce the number of working ports, has been explored as an alternative; however, it brings with it ergonomic issues and surgical complexity while not adding much to the cosmetic outcomes [28, 29, 33, 34].

To address these limitations while preserving the advantages of CLSG, the Bikini-line Sleeve Gastrectomy (BSG) has been introduced as a novel technique. Initially described by Abdelbaki in 2017 [35], BSG modifies the CLSG by repositioning working ports along the bikini line, effectively disguising incisional scars within the natural abdominal folds. This approach aims to enhance cosmetic satisfaction while maintaining the safety and efficacy of the procedure [28, 35, 36].

While BSG offers notable aesthetic improvements, it is critical to acknowledge the potential challenges involved. The lower abdominal port placement necessitates precise anatomical mapping to prevent vascular injury, particularly to the inferior epigastric arteries [37, 38]; failure to adequately identify and safeguard these vessels may lead to intraoperative hemorrhage and hematoma formation. Furthermore, the technical ergonomics of BSG differ significantly from CLSG, requiring adjustments in surgeon positioning, instrument manipulation, and stapler orientation [28, 35, 39]. BSG also involves additional intraoperative evaluations, including hepatic retraction and hiatal assessment, which may contribute to extended operative times, particularly during the initial learning curve [28, 35, 40, 41]. Given the potential benefits and challenges associated with BSG, rigorous investigations are warranted to establish its safety, effectiveness, and impact on patient outcomes compared to the CLSG.

Additionally, while the BSG initially described by Abdelbaki was described as a 4-port procedure, further refinements of this technique were made to enhance the cosmetic integrity of the procedure. This study aims to evaluate the 3-port alternative of BSG, namely the reduced port bikini-line sleeve gastrectomy (RBSG), as a viable alternative to CLSG by examining key parameters, including operative time, intraoperative complications, postoperative pain, and patient-reported satisfaction regarding cosmetic outcomes. Through a systematic comparison of these variables, this study seeks to provide evidence-based recommendations for the integration of RBSG in MBS and to promote the adoption of patient-centered surgical approaches within the domain of MBS.

## Materials and Methods

### Study Design and Ethical Considerations

This study presents a prospective analysis of data collected from patients who underwent 3-port BSG (RBSG) at our institution between September 2022 and September 2023. The research received approval from our institution’s Research Ethics Committee with an approval number N-475–2023, and all participating patients provided written informed consent. The study was conducted following the Declaration of Helsinki [42] and reported in adherence to the Strengthening the Reporting of Observational Studies in Epidemiology (STROBE) guidelines for observational studies [43].

### Patient Selection and Eligibility Criteria

The study comprised adult obese individuals aged 18 to 60 years with a BMI exceeding 40 kg/m^2^ or exceeding 35 kg/m^2^ and have obesity-associated medical problems [44] undergoing elective, primary RBSG. Exclusion criteria included individuals with prior bariatric or upper abdominal surgeries [45, 46], large hiatal hernias [47], patients undergoing concurrent bariatric or non-bariatric surgical procedures, those with a BMI under 35 kg/m^2^, individuals with chronic pain conditions necessitating ongoing opioid therapy, and patients with contraindications to local anesthetic administration [48, 49].

### Sample Size Calculations

The sample size was calculated using [50] for a paired *t*-test (two dependent means). Assuming a medium effect size (dz = 0.5), a significance level (*α* = 0.05), and a statistical power of 80%, the estimated required sample size was 34 patients. To account for a potential 20% dropout rate, the final target sample size was set at 40 patients.

### Participants

**Fig. 1:**
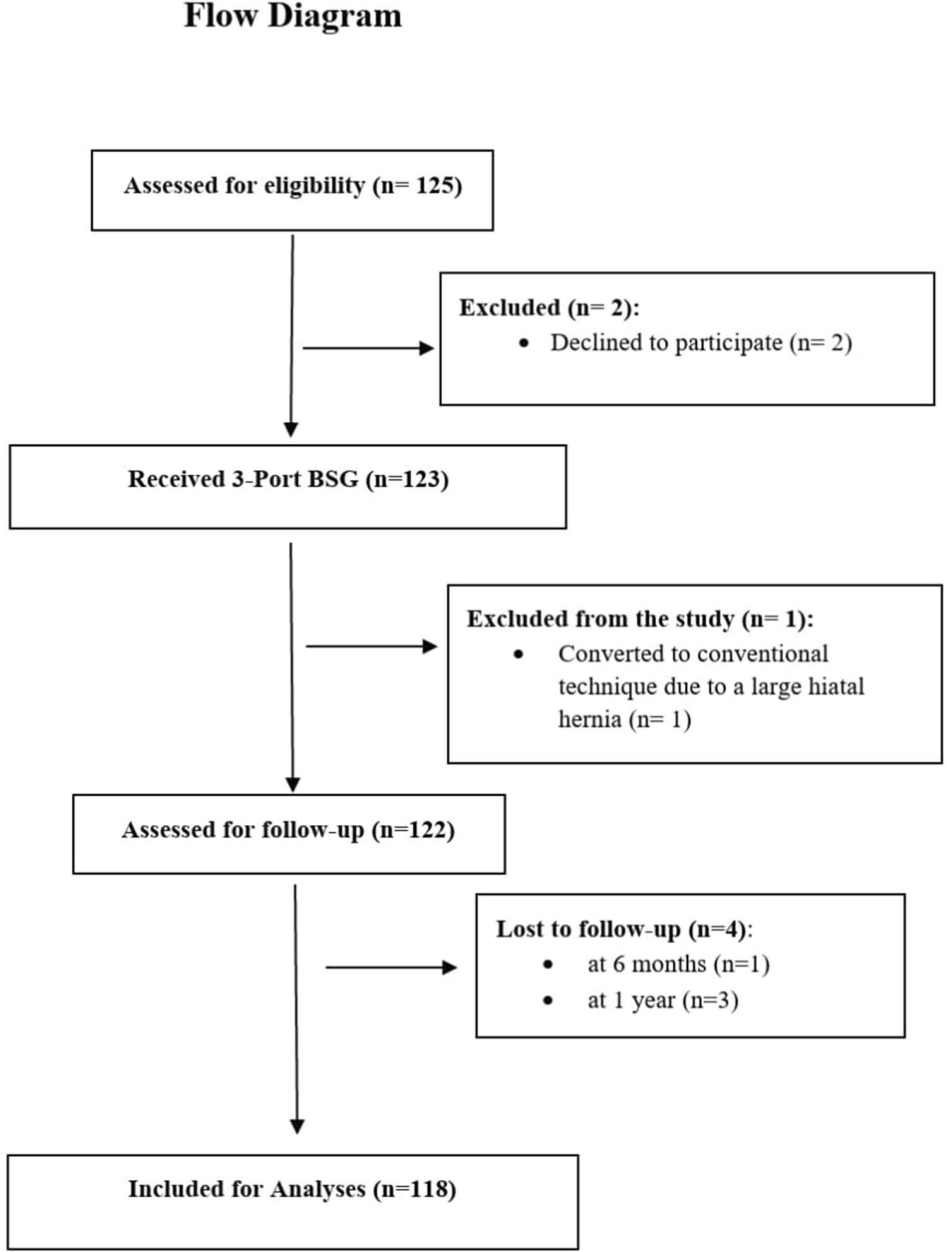
Flowchart of patient selection

Throughout the study period, 125 patients were evaluated for inclusion, with 123 patients ultimately undergoing a 3-port BSG (RBSG) procedure at our facility. Out of this cohort, 5 patients were excluded for various reasons, which are elaborated upon in the flowchart (Fig. 1).

### Data Collection

Data collection occurred at multiple intervals throughout the follow-up period. Preoperative metrics were gathered to establish a baseline, including weight, BMI, associated medical problems, and scores from the body image scale (BIS) [18, 51, 52]. Intraoperative data encompassed the identification of the inferior epigastric artery, the assessment of hiatal hernia presence and size, and any occurrences of gastric specimen rupture during extraction [53], which will later be correlated with postoperative complications.

Postoperative data were obtained at 6 months and 1 year to evaluate complications such as Surgical Site Infections and port site hernias, alongside patient satisfaction and assessments using the BIS. Additionally, weight changes, BMI, and the resolution of associated medical conditions were documented.

### Operative Technique

#### Anesthesia and Perioperative Protocols

All procedures were performed by a single surgeon and a single anaesthesiologist under general anesthesia. Anesthetic induction was achieved using propofol (1.5 mg/kg), fentanyl (1.5 mcg/kg), and atracurium (0.5 mg/kg). A standard ultrasound-guided transversus abdominis plane (TAP) block [54] was administered with 20–30 mL of 0.5% bupivacaine to optimize postoperative analgesia. Dexmedetomidine was infused at 0.5 mcg/kg/h to maintain sedation. Thromboprophylaxis and antibiotic prophylaxis were administered preoperatively.

#### Patient Positioning and Abdominal Entry

The patient was positioned supine with a 15–20° reverse Trendelenburg tilt to optimize visualization of the upper abdomen, with the surgeon standing between the legs. The pneumoperitoneum was established using carbon dioxide insufflation to 15 mmHg, followed by the placement of a 12-mm camera port at the umbilicus. The liver retraction was then achieved via wire insertion at the subxiphoid region to facilitate the assessment of the esophageal hiatus. Once adequate exposure was obtained, an initial exploration was performed to evaluate for the presence of a large hiatal hernia and assess for major adhesions in the lower abdomen and pelvis; either finding would necessitate conversion to a conventional laparoscopic technique.

#### Identification of the Inferior Epigastric Artery

Under visual guidance through the umbilical port, initial exploration is commenced to identify the inferior epigastric artery (IEA). The IEA is either identified via the transillumination method or through anatomical identification of the lateral umbilical ligament laparoscopically.

During the transillumination method, the optical lens light is used to reflect the abdominal vessels in a dim operating room. However, due to the nature of the patient being operated on, the abdominal wall is usually fat, and the identification of the vessels is usually hard to identify. Thus, the anatomical identification method is the only option to make sure to avoid vascular injury.

Although anatomical identification seems safe, it comes with its own challenges. In patients with obesity with their abdomen insufflated, the IEA is usually shifted laterally [55], which poses difficulty in anatomically locating the vessels; however, the identification of the lateral umbilical ligament usually possesses a rough estimate of the location of the IEA if not apparent via the transillumination method, where the IEA is usually medial to the LUI and lateral to the deep inguinal ring [37, 56]. If the vessel is identified by either technique, the trocar is inserted lateral to the vessel. However, if the vessel was not identified, efforts to avoid IEA injury by placing the trocar superior to the anterior superior iliac spine and at least 6–8 cm from the midline at both sides [37].

#### Trocar Placement and Ergonomics

After vascular identification, two trocars are then inserted along the bikini line: a 15 mm trocar is inserted to the left, and a 12-mm trocar is placed on the right side of the bikini line, after identification of the inferior epigastric artery as previously mentioned. This 3-port configuration was designed to optimize triangulation as per Abdelbaki’s technique [35] while minimizing visible scarring.

**Fig. 2:**
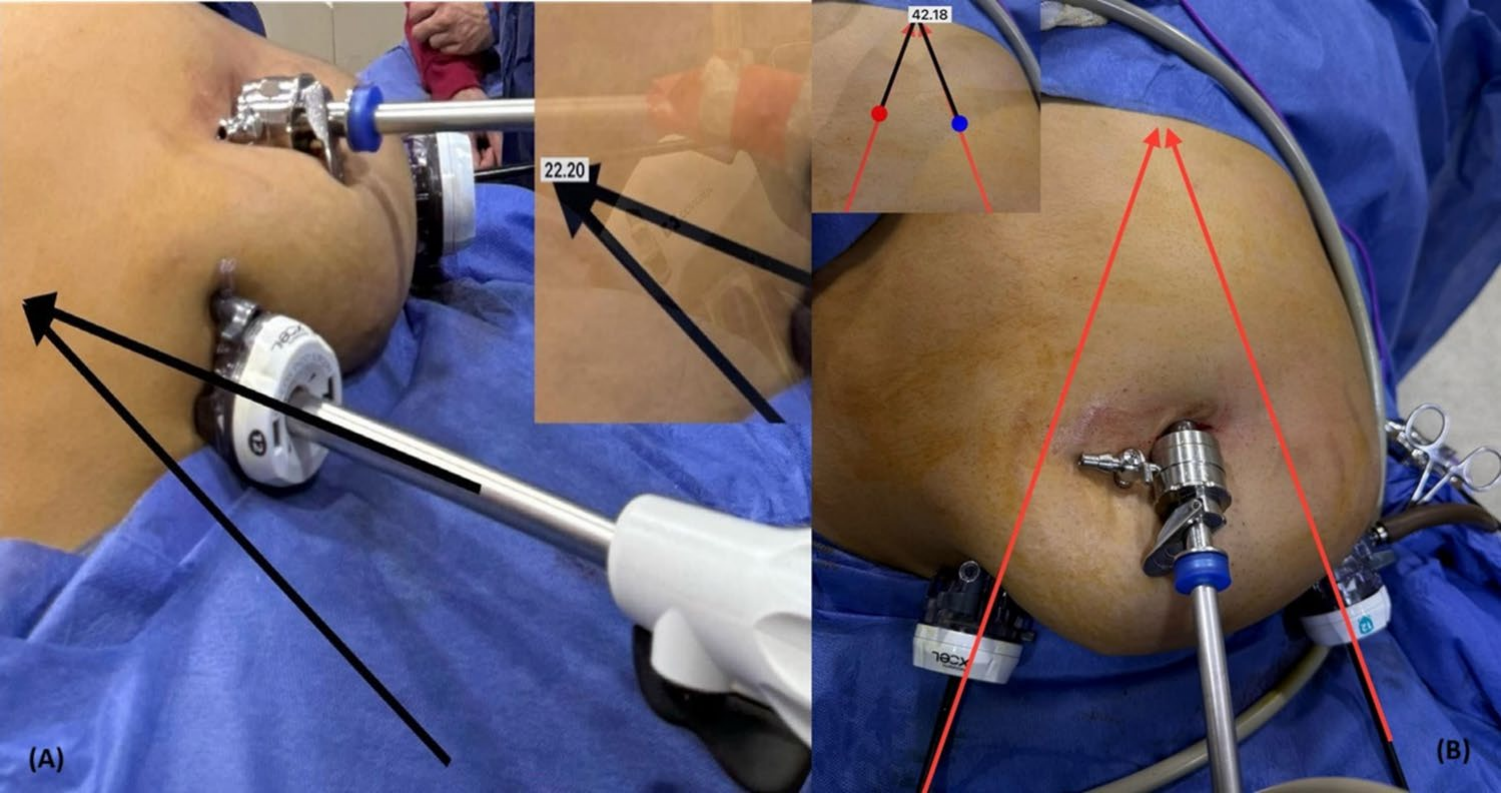
Intraoperative ergonomic assessment of reduced port bikini-line sleeve gastrectomy. (**A**) Elevation angle (22.20°) during gastric dissection. (**B**) Manipulation angle (42.18°) near the hiatus, adjusted to optimize stapler orientation

In terms of ergonomics, the 180 cm tall operating surgeon stands between the legs of the patient, with a moderate azimuth angle of 20–30° used during dissection of the distal stomach (Fig. 2).

#### Gastric Mobilization, Cruroplasty, and Their Ergonomic Challenges

The stomach was mobilized along the greater curvature using a LigaSure™ sealer with Nano-coating [57], starting from the pylorus and extending toward the left crus of the diaphragm. The dissection of the gastric greater curvature extended up to the cardio-esophageal junction. Complete mobilization of the gastric fundus involved dissecting the fat pad to expose the GEJ and identify any small hiatal hernia. Visible signs such as a diaphragmatic defect, GEJ positioned above the diaphragm, or pericardial fat retracted into the hiatus (appearing as a dimple) indicated the presence of a hiatal hernia [41, 58]. If a hiatal hernia was found, a cruroplasty was performed before gastric stapling by placing a traction tape around the esophagus, fully dissecting the diaphragmatic crura to the mediastinum, and reducing the gastric herniation into the abdomen, ensuring at least 3 cm of the intra-abdominal esophagus [41, 59]. The hiatal crural defect was posteriorly repaired using two or more interrupted 2/0 Polyester sutures, and additional anterior sutures were applied to approximate the crura anteriorly.

A 38 Fr bougie was introduced transorally and positioned against the lesser curvature to guide sleeve formation. A linear Endo GIA™ ultra universal stapler [60] is introduced through the left working port. Stapling commenced 4–6 cm from the pylorus and proceeded cranially toward the angle of His.

**Fig. 3:**
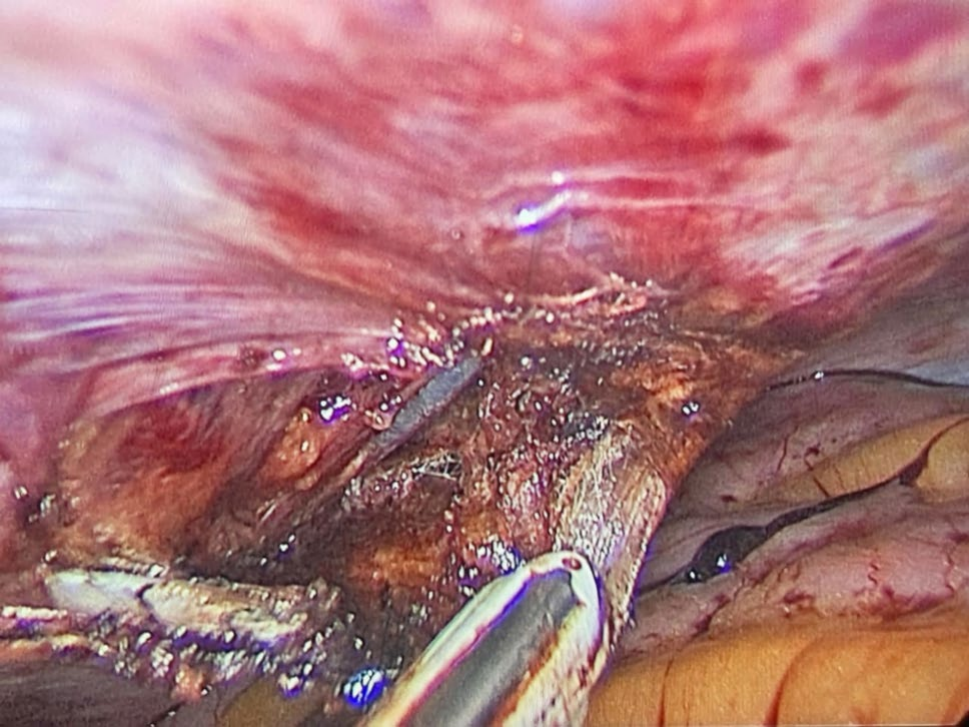
Hemostasis during trocar placement

Stapler manipulation angles were adjusted to optimize traction and tissue handling, minimizing excessive tissue inclusion in the staple line. The surgeon’s manipulation angle of approximately 40–45° (Fig. 2). The azimuth angle, defining the stapler’s orientation relative to the working and elevation axis, was dynamically adjusted (the elevation angle was 22.5° during the application of the last staple) (the elevation angle was measured as two lines meeting one from the stapler, and the other imaginary line represented by the plane of the patient’s body (Fig. 3)). A moderate azimuth angle of 20–30° was used in the distal stomach for gradual orientation, reducing to < 15° near the hiatus [28].

#### Staple Line Reinforcement, Hemostasis, and Wound Closure

Finally, the staple line was reinforced using a 2–0 PDS® II Suture [61], to minimize the risk of leakage and bleeding, and an intraoperative leak test was performed using methylene blue to assess staple line integrity.

**Fig. 4:**
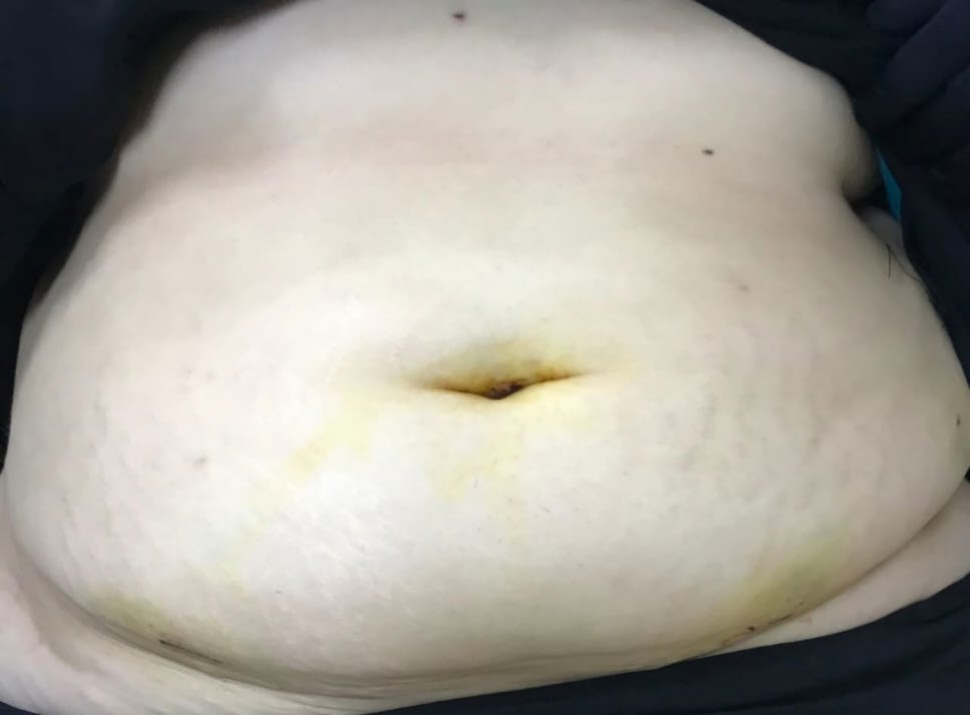
Post-operative image of scarless abdomen

Hemostasis along the staple line was ensured, and Port site homeostasis was done using the LigaSure™ sealer [57] (Fig. 3), and final incisions were meticulously closed to optimize cosmetic outcomes using a 3–0 MONOCRYL® subcuticular suture [62], with scars concealed along the bikini line (Fig. 4).

The primary operating surgeon had previously performed over 4000 CLSG and received dedicated training in the BSG approach. This background supports the technical reproducibility and patient safety reported in this initial series.

#### Postoperative Ward Assessment

All patients underwent postoperative assessment for recovery in the ward. Postoperative pain levels were quantified using a 0–10 numerical rating scale (NRS) [63] at intervals of 6 and 24 h post-surgery, where 0 indicated “no pain” and 10 denoted “the worst pain imaginable.” Patients were encouraged to initiate ambulation as soon as they regained consciousness from anesthesia, with the administration of oral fluids starting 3 h postoperatively. Each patient was discharged 24 h following the surgical procedure.

#### Assessment of Port Site Complications

Port sites were meticulously evaluated for the potential development of wound infections, bleeding, and hernias. During the insertion and extraction of ports, careful attention was given to assessing patients for potential vascular injuries, particularly involving the inferior epigastric arteries. In cases where vascular injuries were identified, advanced bipolar handle techniques were employed to control any resultant bleeding (Fig. 3).

Following the abdominal extraction, gastric specimens were assessed for integrity by applying water inflation to verify their condition. Each specimen was subsequently classified as either “disrupted” or “not disrupted.” All gastric specimens were removed through the right midclavicular trocar positioned at the lowest crease of the abdomen, at the bikini line.

At follow-up, the port sites were thoroughly evaluated for signs of wound infection, hematomas, and development of port-site hernias. Findings were recorded for statistical correlations.

#### Body Image Scale (BIS) Questionnaire

The body image scale (BIS), a validated 9-item questionnaire [64], has been employed and validated in prior MBS studies [18, 52]. In this study, the questionnaire was administered in person by a bilingual research assistant fluent in Arabic and English to ensure comprehension and standardization during patient follow-up. Each item is scored 0–3 (Never to At a Great Deal), with a total possible score range of 0–27.

BIS aimed to assess the impact of RBSG on body image. The questionnaire used in the study was developed and adopted from earlier studies [18, 52, 64]. To mitigate inter-rater variability, one bilingual researcher, fluent in both Arabic and English, administered the questionnaire to all participants.

#### Reliability Analysis

To assess internal consistency of the BIS in our cohort, we calculated Cronbach’s alpha, which yielded a value of 0.68. While this is slightly below the conventional threshold of 0.70, it is considered acceptable for early or exploratory studies, particularly when assessing complex psychological constructs such as body image. This suggests that the BIS demonstrated reasonable consistency in capturing postoperative body image perceptions within our population.

#### Correlation of Hiatal Cruroplasty During RBSG and Operative Time

Concurrent incidental hiatal hernias were identified intraoperatively. Small hiatal hernias were repaired using the same bikini-line ports in alignment with the technique described by Abdelbaki et al. [41]. Operative time was then assessed and compared to those without hiatal hernias. This correlation was done to indirectly assess the ergonomic difficulties encountered during bikini-line ports.

### Statistical Analysis

Data were analyzed using IBM SPSS Statistics 25.0 Fix Pack 1 [65]. Continuous variables are presented as mean ± standard deviation (SD) for data with a normal distribution and as median (interquartile range) for non-normally distributed data. Normality was determined using the Shapiro–Wilk test, complemented by visual assessments of histograms and Q-Q plots.

To evaluate variables across different time points, repeated measures ANOVA was employed for normally distributed data, with Mauchly’s test applied to check for sphericity. In instances where sphericity was not met, the Greenhouse–Geisser correction was employed. For non-normally distributed data, the Friedman test served as the appropriate non-parametric alternative. Post hoc analyses utilized Bonferroni-adjusted pairwise comparisons for the ANOVA results, while the Wilcoxon signed-rank test was used for non-parametric data.

For the comparison of preoperative and postoperative outcomes at each follow-up period, a paired-sample *t*-test was employed for normally distributed data. In cases where the normality assumption was violated, the Wilcoxon signed-rank test was utilized as an alternative. To assess changes in paired binary categorical variables over time, the McNemar test was applied to evaluate preoperative and postoperative outcomes.

In correlation analyses, Spearman’s correlation coefficient was used for non-normally distributed variables, whereas Pearson’s correlation coefficient was applied for normally distributed variables.

To mitigate confounding variables, propensity score matching (PSM) at a 1:1 ratio was used with nearest-neighbor matching without replacement. Variables included in the model were age, sex, and preoperative BMI. Matching was performed using IBM SPSS Statistics 25.0 Fix Pack 1 [65].

PSM was performed on two occasions, The first PSM compared patients with small hiatal hernias to those without, utilizing demographic and baseline clinical characteristics to ensure comparability. The second matching compared patients with and without port site complications, ensuring similar baseline characteristics between the groups.

## Results

### Patient Demographics and Basic Clinical Characteristics

A total of 118 patients participated in the study, with a mean age of 37.76 years (± 12.08). The cohort was predominantly female (*n* = 79, 66.9%). The average BMI among participants was 47.16 ± 5.98 kg/m^2^. The mean operative duration was 48.97 ± 8.51 min, ranging from 38 to 90 min. Intraoperatively, an incidental diagnosis of small hiatal hernia was observed in 31.4% of cases (Table 1).

**Table 1:** Demographics and operative characteristics of the study population (*n* = 118)

	*n* = 118 *F* (%)
Demographics and basic characteristics	
Age (years)	
Mean ± SD	37.76 ± 12.08
Range	19–70
Sex	
Male	39 (33.1)
Female	79 (66.9)
BMI (Kg/m^2^)	
Mean ± SD	47.16 ± 5.98
Range	36.29–68.13
DM	21 (17.8)
HTN	12 (12.2)
Peri-operative characteristics	
Operative time (min)	
Mean ± SD	48.97 ± 8.51
Range	38–90
Inferior epigastric artery (IEA)	
IEA was identified by locating the lateral umbilical ligament	117 (99.2)
IEA was identified via the transillumination method	40 (33.9)
IEA injury	2 (1.7)
Incidentally diagnosed with a small hiatal hernia	37 (31.4)
Gastric specimen violation during extraction	6 (5.1)
Post-operative complication	
SSI at the bikini line on the 7th postoperative day	
Yes	4 (3.4)
No	114 (96.6)
Leakage	1 (0.8)

Notably, staple-line leakage was reported in one patient (0.8%), leading to re-admission and necessitating laparoscopic exploration via new upper abdominal wall ports, peritoneal lavage, and concurrent endoscopic Mega-stent placement.

Preoperative weight averaged 119.59 ± 19.05 kg, significantly decreasing to 90.79 ± 11.51 kg at 6 months and further to 80.06 ± 9.54 kg at 1 year (*p* < 0.001). Total weight loss (TBWL%) was recorded at 23.65 ± 3.88% at 6 months and 32.54 ± 4.53% at 1 year (*p* < 0.001). Excess weight loss (EWL%) was recorded at 51.10 ± 4.45% at 6 months and 75.24 ± 5.5% at 1 year (*p* = 0.002) (Supplementary File 1, Table S1).

Notably, HbA1c levels demonstrated significant improvement, decreasing from 8.87 ± 0.47% preoperatively to 5.86 ± 0.54% at 6 months and 5.39 ± 0.45% at 1 year (*p* < 0.001). Before the intervention, 10.2% of the cohort had a diagnosis of hypertension, which was reduced to 5.9% at the one-year mark, although this change did not reach statistical significance (*p* = 0.063) (Supplementary File 1, Table S2).

### Port-Site Placement–Related Complications

IEA injuries occurred in 1.7% of cases during port site insertion, all of which were effectively managed using advanced bipolar coagulation (Table 1) (Fig. 3). Port site hernias were observed in 1.7% of patients at both the 6-month and 1-year follow-up. Notably, none of the patients with IEA injuries developed a port-site hernia at the 3- or 6-month intervals, denoting no correlation between IEA injuries and port-site hernias.

Additionally, gastric specimen disruption during extraction was reported in 5.1% of cases. Surgical site infections (SSIs), specifically at the bikini line, were identified in four patients, accounting for 3.4% (*n* = 4) of the cohort. Notably, only one case of gastric specimen disruption was linked to an SSI.

### Body Image Scale (BIS)

The postoperative evaluation of body image indicated a statistically significant reduction in the overall BIS scores, which declined from an average of 18.58 ± 1.87 preoperatively to 11.44 ± 1.99 postoperatively (*p* < 0.001). Assessment of the BIS constituents revealed significant enhancements in patients’ satisfaction with their physical appearance and overall comfort regarding body image (Table 2).

**Table 2:** Preoperative vs. postoperative body image scale (BIS) scores

	Pre-operative BIS	Post-operative BIS	*p*-value
Mean ± SD	18.58 ± 1.87	11.44 ± 1.99	< 0.001
Min–max range	14–23	7–17	

After employing propensity score matching (1:1) to account for port site complications while controlling demographic variables such as age and BMI, no significant differences in BIS scores were found between patients experiencing port site complications (which encompassed bleeding, infection, or port site hernias) and those who did not (*p* = 0.290). This finding indicates a lack of a direct relationship between the occurrence of port site complications and BIS scores (Table S5).

Further analysis revealed that the BIS post-operative scores did not vary significantly concerning gender or age. Males exhibited a median BIS score of 11 (IQR: 10–12), whereas females had a median score of 12 (IQR: 10–13) (*p* = 0.161). Participants aged 30 years or younger had a median score of 12 (IQR: 10–13), in contrast to a median of 11 (IQR: 10–12) for individuals older than 30 years (*p* = 0.810). Pearson’s correlation analysis did not find any significant association between post-operative BIS scores and TBWL% at 6 months (*r* = 0.131, *p* = 0.203) or 12 months (*r* = 0.120, *p* = 0.245), nor between post-operative BIS scores and EWL% at 6 months (*r* = 0.163, *p* = 0.112). However, a significant negative correlation was observed between the post-operative BIS scores and EWL% at 12 months (*r* =  − 0.486, *p* =  < 0.001), suggesting that patients with greater EWL% are associated with improved body image perception (Table 3).

**Table 3:** Analysis of BIS scores to demographics, surgical site infection (SSI), and BMI

	Post-operative body image scale	*p*-value
Sex		0.161
Male	11 (10–12)	
Female	12 (10–13)	
Age		0.810
Less than or equal to 30 years	12 (10–13)	
More than 30 years	11 (10–12)	
SSI		0.661
With	11 (10–11)	
Without	11 (10–13)	
Inferior epigastric artery injury	11.0 ± 0.0	0.756
Gastric specimen violation during extraction	13.40 ± 1.52	**0.023**
Port site hernia	11.50 ± 0.71	0.965
EWL% (6 months)	*r*(*p*)	
	0.163 (0.112)	
EWL% (12 months)	*r*(*p*)	
	*− 0.486* (**< 0.001**)	
TBWL% (6 months)	*r*(*p*)	
	*0.131 (0.203)*	
TBWL% (12 months)	*r*(*p*)	
	*0.120 (0.245)*	

Median (IQR), mean ± SD, *r*(*p*) = Pearson’s correlations

IEA injuries demonstrated no significant correlation with BIS scores, evidenced by a *p*-value of 0.756 (Table 3). Conversely, patients who encountered gastric specimen violations during extraction exhibited markedly elevated BIS scores, averaging 13.40 ± 1.52, reflecting a deterioration in body image perception. This observed discrepancy was statistically significant, with a *p*-value of 0.023 (Table 3).

Interestingly, when evaluating the relationship between post-operative BIS scores and the presence of surgical site infections (SSI), no significant differences emerged. The median BIS for patients with SSI was 11 (IQR: 10–11), compared to 11 (IQR: 10–13) for those without SSI, yielding a *p*-value of 0.661 (Table 3).

Additionally, an assessment of port site hernias revealed no significant variations in BIS scores at 6 and 12 months postoperatively, with *p*-values of 0.965 recorded at both intervals (Table 3).

### The Effect of Incidental Hiatal Hernial Repair and Operative Time

**Fig. 5:**
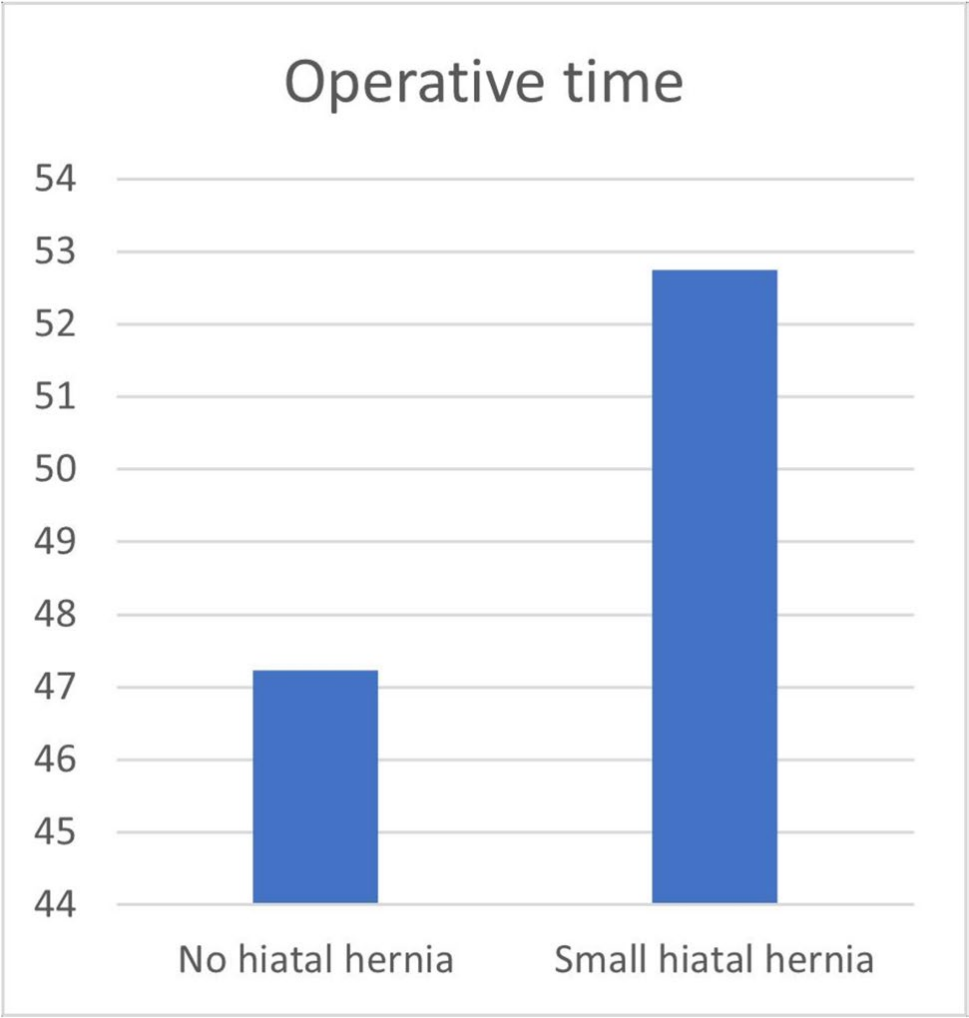
Operative time comparison

Following 1:1 propensity score matching to control for confounding variables such as age and BMI, we analyzed operative duration in patients with small hiatal hernias compared to those without. The results indicated that the median operative time for patients with small hiatal hernias was significantly longer, at 50 min (IQR: 46.5–56), in contrast to 45 min (IQR: 43–49) for the control group (*p* = 0.001) (Table 4) (Fig. 5). However, an assessment of weight loss outcomes between the two cohorts showed no statistically significant difference in BMI at 6 months (*p* = 0.922) or 12 months (*p* = 0.469). Similarly, EWL% at 6 months (*p* = 0.236) and 12 months (*p* = 0.693) did not differ significantly between the groups. TBWL% at 6 months and 12 months also showed no significant differences between groups (*p* = 0.268, *p* = 0.341, respectively) (Table 4).

**Table 4:** Operative time and weight loss outcomes in patients with vs. without hiatal hernia after propensity score matching (PSM)

	**Small hiatal hernia**	**No hiatal hernia**	***p*-value**
	**(*n* = 37)**	**(*n* = 37)**	
Operative time, min	50 (46.5–56)	45 (43–49)	**0.001**
BMI, 6 months	35.17 (33.22–37.56)	35 (34.02–36.6)	0.922
BMI, 12 months	31.13 (30.17–32.56)	31.38 (30.5–32.4)	0.469
EWL%, 6 months	50.45 (49.09–53.2)	52.08 (48.7–53.7)	0.236
EWL%, 12 months	75.14 (72.5–79.8)	75.1 (72.5–76.5)	0.693
TBWL%, 6 months	22.73 (20.87–25.46)	23.57 (21.51–26.49)	0.268
TBWL%, 12 months	31.49 (28.55–34.74)	32.19 (29.71–34.94)	0.341

### Correlations with Pain (NRS Score)

Pain scores assessed at 6 h (NRS-6 h) and 12 h (NRS-12 h) were correlated with various operative and clinical parameters. There was no significant correlation between pain scores and IEA injuries at 6 h (*p* = 0.345) or 12 h (*p* = 0.768) (Table 5).

**Table 5:** Correlations between operative time, iea injury, pain scores, and postoperative body image (BIS)

		Inferior epigastric artery injury	Operative time	BIS
NRS (6 m)	**Median** **Q1**–**Q3** ***p*-value**	1 (1–1) 0.345	*r*(*p*) − 0.270 (**0.003**)	*r*(*p*) − 0.107 (0.300)
NRS (12 m)	**Median** **Q1**–**Q3** ***p*-value**	1 (1–1) 0.768	*r*(*p*) 0.105 (0.257)	*r*(*p*) 0.334 (**0.001**)

*p*-value = Mann–Whitney test

*r*(*p*) = Spearman correlations

A weak but significant negative correlation was noted between operative time and pain scores at 6 h (*r* =  − 0.270, *p* = 0.003), suggesting that longer operative times might be associated with reduced pain. However, this correlation was not evident at 12 h (*r* = 0.105, *p* = 0.257) (Table 5).

Regarding BIS scores, no significant correlation existed between BIS and NRS at 6 h (*r* =  − 0.107, *p* = 0.300), but at 12 h, a weak positive correlation emerged (*r* = 0.334, *p* = 0.001), indicating that poorer body image perception correlated with higher pain levels (Table 5).

## Discussion

Bikini-line sleeve gastrectomy (BSG) has emerged as a promising alternative to CLSG by repositioning ports at the lower abdomen to improve aesthetic outcomes. This study evaluated a novel 3-port variation of the BSG technique, RBSG, to determine whether it retains the metabolic benefits of CLSG while reducing scarring in the upper abdomen. Our findings indicate that RBSG may offer weight loss results and safety profiles comparable to conventional methods, along with the potential added benefit of improved body image as reported by patients.

### Technical Feasibility and Operative Outcomes

A key concern regarding new port placement strategies is whether altering surgical access impacts technical feasibility or leads to extended operative times. In this study, the average procedure duration was 48.97 ± 8.51 min, which is well within the typical range for CLSG, usually reported as approximately 40 to 60 min in many experienced centers [19, 20]. Patients presenting with incidental small hiatal hernias demonstrated significantly prolonged operative times; however, this variation is understandable given the additional surgical procedures involved in hiatal hernia reduction and cruroplasty. The data suggest that relocating working ports to the bikini line does not significantly elevate the technical complexity of the surgical procedure. This observation is consistent with prior studies indicating that the effective implementation of reduced-port or single-incision techniques considerably relies on the surgeon’s proficiency and the requisite ergonomic adjustments made during the operation to maintain both efficiency and precision [35, 66–68]. By refining surgical techniques to align with the surgeon’s skills, it is possible to achieve favorable outcomes without jeopardizing patient safety or the overall effectiveness of the surgery [27–29, 68].

**Fig. 6:**
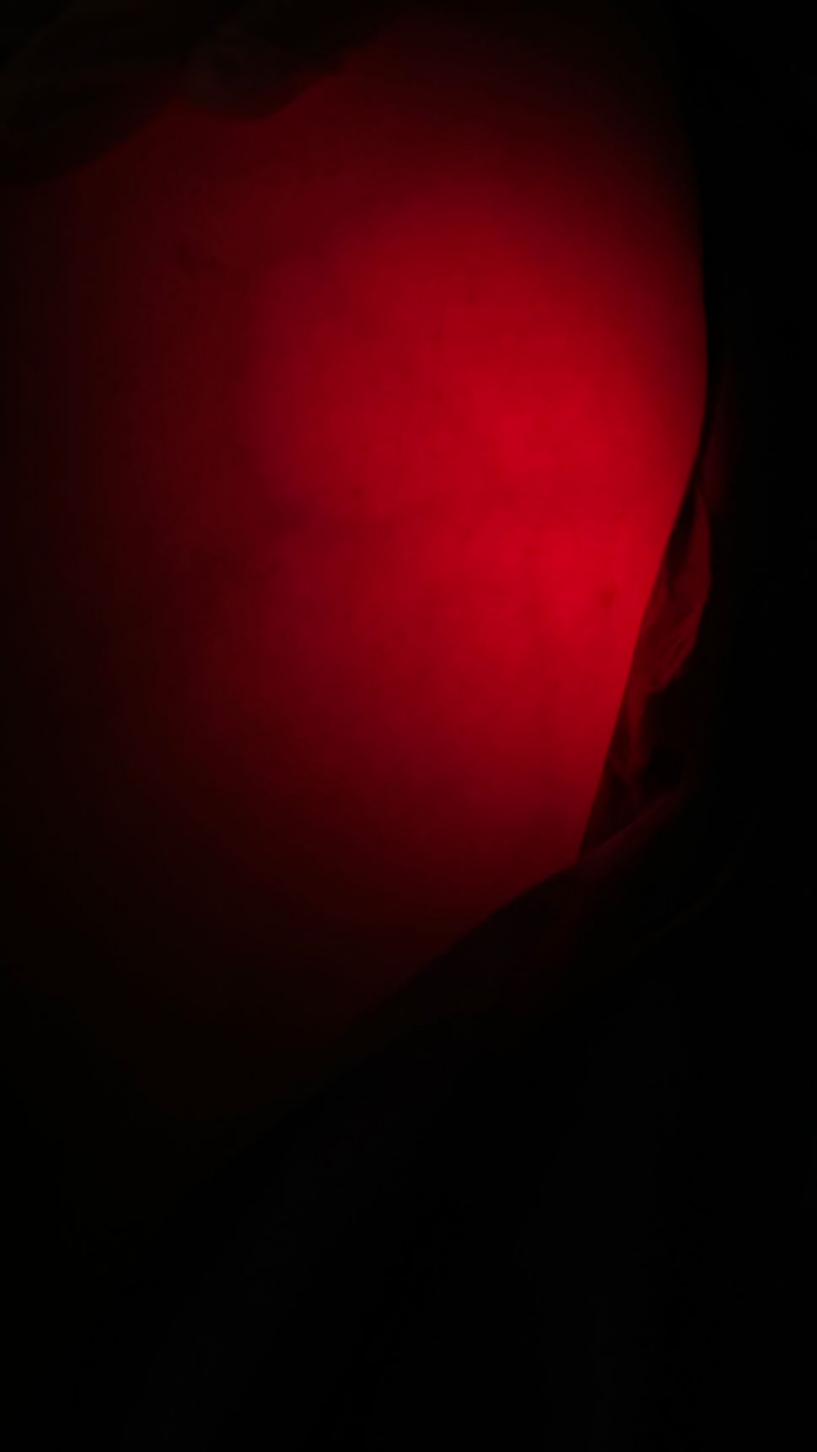
Transillumination of IEA

In our cohort, 1.7% of the patients experienced inferior epigastric artery (IEA) injuries during port placement (Fig. 3). While rare, IEA injuries can occur in any laparoscopic technique, particularly when ports are positioned in the lower abdominal region [30, 37, 38]. Notably, all IEA injuries were controlled intraoperatively using advanced bipolar coagulation without further sequelae (Fig. 3). This underscores the importance of identification of the inferior epigastric vessels via trans-illumination (Fig. 6) and detailed anatomical familiarity—particularly important when adopting an alternative port site approach [37, 55].

### Body Image, Cosmesis, and Patient-Centered Outcomes

A central rationale for repositioning the ports along the bikini line is the potential to improve cosmetic results, thus improving patients’ image perception. The marked decline in BIS scores in our findings suggests that RBSG may positively impact patients’ perceptions of their bodies. This improvement suggests that the bikini-line approach may offer psychological benefits related to reduced scarring. However, the absence of a significant statistical correlation between BIS scores and weight loss, alongside a weak correlation between BIS scores and postoperative pain at 12 h, underscores the multifactorial nature of body image perception [69].

This improvement is especially noteworthy given that many MBS patients, although satisfied with weight loss, can experience lingering dissatisfaction related to surgical scars or excess skin [18, 25, 26]. This finding reinforces the idea that aesthetic factors, such as scar placement, may independently contribute to enhanced postoperative body image, thereby complementing the psychosocial benefits associated with weight reduction [18, 51, 52].

Although this study does not directly compare RBSG to CLSG or to other techniques, postoperative BIS scores denoted in this study seem to be notably lower when compared to other studies. For instance, Abokhozima et al. [18] reported postoperative BIS scores of 14.9 ± 3.1, which are notably higher than our RBSG cohort (11.44 ± 1.99), suggesting enhanced aesthetic satisfaction with the bikini-line approach.

Moreover, our analysis indicates that female patients in our cohort exhibited slightly higher postoperative BIS scores than their male counterparts, corroborating literature that highlights increased sensitivity to body image issues among women [70, 71]. These results underscore the necessity for tailored counseling and gender-sensitive outcome assessments in future research, particularly regarding scarring and aesthetic considerations [72, 73].

Conversely, we found no significant age-related differences in BIS scores (*p* = 0.861), suggesting that the cosmetic advantages of RBSG may be perceived similarly across various age demographics. However, this conclusion should be approached with caution, as prior studies have demonstrated a significant decline in body image perception with advancing age [74], influenced by cultural and socioeconomic variables. Therefore, generalizing our findings is not appropriate, considering that body image is intricately linked to multiple factors, including psychiatric, socioeconomic, regional, and ethnic dimensions [69, 75].

Compared to SISG, which has been linked to challenges such as higher incisional hernia rates, port crowding, and potential for greater postoperative pain [30, 31], the RBSG approach seems to achieve similar cosmetic gains but with lower technical demands. While SISG may offer a nearly scarless abdomen, large fascial defects at the umbilicus may predispose patients to port-site complications [31]. In contrast, a well-placed bikini-line port configuration might show a favorable balance between reduced scarring and manageable operative ergonomics.

### Operative Time and Postoperative Pain

Operative time constitutes a significant consideration in the context of MBS, particularly when evaluating novel surgical approaches. Our data revealed an average operative duration, indicating that the repositioning of surgical ports to the bikini line does not substantially prolong surgical duration.

The relationship between operative time and postoperative pain offers valuable insights into the complex nature of pain perception following MBS procedures. Our research demonstrated a weak yet significant negative correlation between operative time and immediate postoperative pain measured on the NRS scale at 6 h post-surgery (*r* =  − 0.270, *p* = 0.003). This inverse relationship suggests that an extended surgical duration, when executed with care and precision, may not necessarily increase postoperative pain. This finding contrasts with the conventional understanding found in the literature, which typically posits that longer operative times correlate with higher levels of postoperative pain [76]. Our findings might suggest that a meticulous dissection might minimize tissue trauma and reduce excessive retraction, which could mitigate postoperative inflammatory responses and subsequent pain perception.

Moreover, the impact of operative time on postoperative pain seems multifactorial and not solely dependent on procedural duration. Factors such as the number of trocars, surgical technique, intraoperative analgesic protocols, and patient positioning can significantly influence postoperative pain experiences. Additionally, reducing the number of trocar sites in RBSG likely contributes to a lower cumulative nociceptive stimulus compared to traditional five-port laparoscopic procedures, although evidence regarding this correlation remains controversial [33].

Postoperative pain assessed using the NRS was overall low, reflecting effective analgesic management of the TAP block and favorable ergonomics of the bikini-line approach. Pain scores remained consistently low both at 6 and 12 h after surgery, demonstrating minimal patient discomfort postoperatively. Furthermore, the absence of a significant correlation between IEA injuries and postoperative pain reinforces the safety and analgesic effectiveness of this surgical technique.

Interestingly, a weak but statistically significant positive correlation was observed between 12-h postoperative pain levels and poorer body image perception, underscoring the psychological dimensions that may influence early postoperative experiences [69]. This relationship suggests that patient-specific psychological factors—such as pre-existing concerns about body image, anxiety, or apprehension about scarring—may shape the perception of pain, independent of purely surgical or physiological variables [72, 77]. Moreover, the moderate correlation between 12-h NRS scores and postoperative BIS outcomes supports the notion that postoperative pain may transiently impair self-perception, confidence, or emotional adjustment [78].

### Complications

Overall, the complication profile in this series was low, reflecting the established safety of RBSG [6, 7]. One patient (0.8%) experienced staple line leakage near the gastroesophageal junction. The patient was managed with resuscitation followed by laparoscopic exploration using newly placed right and left upper abdominal wall ports, which allowed for ergonomically feasible peritoneal lavage and drainage of various intra-abdominal collections. The overall leak rate remains comparable to standard laparoscopic sleeve gastrectomy techniques [79, 80].

Port-site hernias occurred in only 1.7% of patients over a one-year follow-up, a rate consistent with standard multi-port laparoscopic approaches [28]. Similarly, SSIs were observed in 3.4% of patients, aligning with typical rates reported for MBS [53]. Gastric specimen disruption was noted in 5.1% of cases, and although these events did not cause major morbidity, they correlated with higher postoperative BIS scores, reinforcing the connection between operative complications and diminished body image perception. This can be explained by the fact that operative complications such as specimen disruption eventually lead to an increased probability of wound infection, which consequently impacts wound healing and scar shape [81].

Our findings conclude that the significant aesthetic and psychological improvements did not come at the expense of clinical efficacy, as evidenced by BMI reduction and low complication rates. These findings suggest that RBSG may offer a viable alternative to CLSG, particularly in patient populations placing a high value on postoperative cosmesis and body image.

## Strengths and Limitations

This study presents several noteworthy strengths. It is the first to prospectively assess both clinical and aesthetic outcomes of reduced port bikini-line sleeve gastrectomy (RBSG). By utilizing validated assessment tools such as the body image scale (BIS) and employing robust statistical methodologies, including propensity score matching, the study achieves a commendable level of internal validity. Furthermore, the integration of objective surgical outcomes alongside subjective patient-reported metrics reflects a comprehensive, patient-centered framework for metabolic and bariatric surgery (MBS) care. This highlights the growing importance of aesthetic and psychosocial considerations in evaluating the success of enhanced recovery protocols. Importantly, the final study cohort exceeded the pre-determined sample size, enhancing statistical power and reinforcing the generalizability of findings to comparable clinical environments.

However, several limitations warrant consideration. Firstly, the study was conducted at a single high-volume center, with all procedures performed by a single surgeon. While this strengthens internal consistency, it may limit external generalizability. The learning curve associated with alternative port placement strategies, such as those used in RBSG, may also impact reproducibility in centers without equivalent surgical expertise [28, 35]. To aid reproducibility, details of the lead surgeon’s background, including prior experience have been discussed.

Secondly, the absence of a direct comparator group of patients undergoing conventional multi-port laparoscopic sleeve gastrectomy (CLSG) or single-incision sleeve gastrectomy (SISG) restricts the ability to draw definitive comparative conclusions. This limitation arose because RBSG had become the default primary procedure at our institution during the study period. Nevertheless, to partially mitigate this, we included BIS data from published CLSG cohorts to contextualize our findings.

Thirdly, while the 12-month follow-up period is sufficient for assessing early weight loss, complication rates, and body image perception, it does not allow for evaluation of long-term durability. Perceptions of body image are dynamic and may evolve beyond the first year as patients experience new psychosocial challenges, including the impact of skin redundancy or weight regain [18, 82–84]. Although early improvements in body image may be driven by rapid weight loss and the near-invisible scar placement of RBSG, long-term satisfaction may be influenced by factors that emerge well beyond the initial postoperative phase. Future longitudinal studies with extended follow-up are necessary to capture these changes and establish the enduring efficacy and psychosocial benefits of the RBSG approach.

Lastly, although the BIS is a validated instrument frequently employed in MBS studies [52], it remains a subjective self-report measure that can be influenced by individual expectations, cultural norms, and psychological conditions that are not fully captured within the scope of this study. Although the BIS remains a subjective, patient-reported measure, the internal consistency within our cohort (Cronbach’s alpha = 0.68) was acceptable for exploratory psychosocial evaluation.

## Future Directions

To deepen our understanding of aesthetic-focused MBS approaches, future multicenter, randomized trials with extended follow-up periods are essential. These studies should evaluate the long-term sustainability of improvements in body image perceptions and identify emerging differences in port-site complications and rates of reoperations. Additionally, examining the learning curve associated with RBSG and BSG is critical. This includes analyzing trends in operative times and complication rates across various centers to determine their broader applicability in different clinical contexts.

Moreover, a detailed investigation into patient-reported outcomes is warranted, employing validated metrics for quality of life, self-esteem, and psychosocial functioning.Specifically, Our study did not include specific physical performance assessments or detailed body composition analyses such as Bioelectrical Impedance Analysis (BIA). Incorporating these objective measures in future investigations would provide a more comprehensive understanding of the holistic impact of RBSG on patient health and well-being beyond weight loss and body image perception. Such an approach would offer a more holistic view of the benefits associated with these surgeries. As there is an increasing focus on patient-centered care in MBS, these insights could be pivotal in driving enhancements in surgical protocols and practices.

## Conclusion

RBSG appears to be a safe and effective modification of CLSG that preserves metabolic benefits while significantly improving patients’ perceptions of their postoperative physiques. Low rates of port-site complications, robust weight loss, and enhanced body image support the feasibility of this approach. Larger, randomized studies with extended follow-up are warranted to determine the broader applicability of RBSG and to elucidate long-term patient-centered outcomes. By integrating aesthetic considerations with surgical efficacy, RBSG may encourage more patients to opt for bariatric surgery, ultimately expanding access to a life-changing intervention for obesity and its associated medical conditions.

## Supplementary Information

Below is the link to the electronic supplementary material.

Supplementary file1 (DOCX 650 KB)

## Data Availability

Data is provided within the manuscript or supplementary information files.
