# Correction: Assessing the Safety and Aesthetic Benefits of Reduced Port Bikini-Line Sleeve Gastrectomy (RBSG): an Initial Report

**DOI:** 10.1007/s11695-026-08532-5

**Published:** 2026-02-16

**Authors:** Abdelkarem Ahmed Abdelkarem Mohamed, Mostafa Mahmoud Abdelfatah, Mohamed Nasr Shazly, Mina Adolf Helmy, Ahmed Nasr Shazly, Mohamed Saber Mostafa

**Affiliations:** 1 General Surgery Department ‑ Kasralainy School of Medicine, Cairo University, Giza, Egypt; 2 Anesthesia Department, Armed Forces Hospital, Southern Region, Saudi Arabia; 3 Anesthesia Department, Ahmed Maher Teaching Hospital, Cairo, Egypt; 4 Anesthesia Department ‑ Kasralainy School of Medicine, Cairo University, Cairo, Egypt

**Correction: Obesity Surgery (2025) 35:3491-3506**

10.1007/s11695-025-08176-x

The published article states that the study was conducted between September 2022 and September 2023.

The correct study period is September 2023 to December 2024.

This was a clerical error during manuscript preparation and does not affect the data, results, analysis, or conclusions of the study.

