# Supplementary material for: Assessing the Safety and Aesthetic Benefits of Reduced Port Bikini-Line Sleeve Gastrectomy (RBSG): An Initial Report
Source: Obes Surg. 2025 Aug 20;35(9):3491–506. doi: 10.1007/s11695-025-08176-x (PMC12457456; doi:10.1007/s11695-025-08176-x)

# Supplementary File 1

**Table S1**: Key weight outcomes: Percentage of excess weight loss (%EWL), total weight loss, Body Mass Index (BMI) changes from baseline to 6 months postoperatively, and incidence of port-site hernia.

|  | | **Pre-operative** | **Follow-up** | | **P-value** |
| --- | --- | --- | --- | --- | --- |
|  |  |  | **6 Months** | **1 Year** |  |
| **BMI** | **Mean ± SD**  **Range** | 47.16 ± 5.98  36.29 – 68.13 | 35.82 ± 3.01  31.21 – 46.61 | - | **<0.001** |
| **Weight lost** | **Mean ± SD**  **Range** | - | 28.81 ± 8.78  14 – 60 | 42.13 ± 11.78  22 – 80 | **<0.001** |
| **EWL%** | **Mean ± SD**  **Range** | - | 51.10 ± 4.45  33.71 – 71 | 75.24 ± 5.5  62.43 – 92.17 | **0.002** |
| **TBWL%** | **Mean ± SD**  **Range** | - | 23.65 ± 3.89  14 – 37.93 | 32.54 ± 4.53  21.78 – 46.9 | **<0.001** |
| **Port site hernia** | **Yes**  **No** | - | 2 (1.7)  116 (98.3) | 2 (1.7)  116 (98.3) | 1.000 |

**Table S2:** Changes in Body Weight, Glycemic Control, HTN, and Numeric Rating Scale (NRS) Over Time

|  | | **Pre-operative** | **Follow-up** | | **P_2_** |
| --- | --- | --- | --- | --- | --- |
|  |  |  | **6 Months** | **1 Year** |  |
| **Weight** | **Mean ± SD**  **Range** | 119.59 ± 19.05  85– 190 | 90.79 ± 11.51  67 – 130 | 80.06 ± 9.54  59.80 – 105.81 | <0.001 |
|  | **P_1_** | - | <0.001 | <0.001 |  |
| **HbA1c** | **Mean ± SD**  **Range** | 8.87 ± 0.47  7.9– 9.5 | 5.86 ± 0.54  5 – 7 | 5.39 ± 0.45  5 – 6.5 | 0.02 |
|  | **P_1_** | - | <0.001 | <0.001 |  |
| **HTN** | **N (%)** | 12 (10.2) | **-** | 7 (5.9) | 0.063 |
|  |  |  | **6 hours** | **24 hours** |  |
| **NRS score** | **Median**  **Q1 – Q3**  **Range** | - | 1  1 – 2  1 – 3 | 1  1 – 1  1 – 2 | - |

**P1: adjusted Bonferroni p-value for ANOVA with repeated measures for comparison between preoperative and each other period of follow up (over time)
P2: Compare between preoperative and at end of follow-up.**

**Table S3:** Body Image Scale among participants pre-operatively (n=96)

| Body Image Scale (Pre-operative) | At a great deal  F(%) | Sometimes  F(%) | Rarely  F(%) | Never  F(%) | Total score |
| --- | --- | --- | --- | --- | --- |
|  |  |  |  |  |  |
| Are you self-conscious about your appearance | 57 (59.4) | 35 (36.4) | 3 (3.13) | 0 (0) | 2.57 ± 0.56 |
| Have you felt less physically attractive because of your weight? | 73 (76) | 23 (24) | 0 (0) | 0 (0) | 2.76 ± 0.43 |
| Are you dissatisfied with the way you look when you get dressed? | 0 (0) | 66 (68.8) | 28 (29.2) | 2 (2.1) | 1.67 ± 0.52 |
| Do you feel less feminine or masculine because of your weight? | 0 (0) | 38 (39.6) | 29 (30.2) | 29 (30.2) | 1.09 ± 0.83 |
| Do you find it difficult to look at yourself naked (without clothes)? | 38 (39.6) | 50 (52.1) | 8 (8.3) | 0 (0) | 2.31 ± 0.62 |
| Have you felt less sexually attractive because of your weight? | 0 (0) | 74 (77.1) | 22 (22.9) | 0 (0) | 1.77 ± 0.42 |
| Do you avoid people because of how you feel about your appearance? | 0 (0) | 52 (54.2) | 42 (43.8) | 2 (2.1) | 1.52 ± 0.54 |
| Do you feel that obesity has made your body less full? | 62 (64.8) | 34 (35.4) | 0 (0) | 0 (0) | 2.65 ± 0.48 |
| Have you ever felt dissatisfied with your body? | 26 (27.1) | 70 (72.9) | 0 (0) | 0 (0) | 2.27 ± 0.45 |
| Total score | 18.58 ± 1.87 | | | | |

**Coding: At great deal = 3 , Sometimes = 2 , Rarely = 1 , Never = 0**

**Table S4:** Body Image Scale among participants post-operatively (n=96)

| Body Image Scale (Post-operative) | at a great deal  F(%) | Sometimes  F(%) | Rarely  F(%) | Never  F(%) | Total score |
| --- | --- | --- | --- | --- | --- |
|  |  |  |  |  |  |
| Are you self-conscious about your appearance | 57 (59.4) | 35 (36.4) | 3 (3.13) | 0 (0) | 2.57 ± 0.56 |
| Have you felt less physically attractive because of your weight? | 0 (0) | 23 (24) | 73 (76) | 0 (0) | 1.24 ± 0.43 |
| Are you dissatisfied with the way you look when you get dressed? | 0 (0) | 31 (32.3) | 63 (65.6) | 2 (2.1) | 1.30 ± 0.51 |
| Do you feel less feminine or masculine because of your weight? | 0 (0) | 2 (2.1) | 29 (30.2) | 65 (67.7) | 0.34 ± 0.52 |
| Do you find it difficult to look at yourself naked (without clothes)? | 1 (1) | 70 (72.9) | 25 (26) | 0 (0) | 1.75 ± 0.46 |
| Have you felt less sexually attractive because of your weight? | 0 (0) | 47 (49) | 49 (51) | 0 (0) | 1.49 ± 0.50 |
| Do you avoid people because of how you feel about your appearance? | 0 (0) | 12 (12.5) | 40 (41.7) | 44 (45.8) | 0.67 ± 0.69 |
| Do you feel that obesity has made your body less full? | 1 (1) | 71 (74) | 15 (15.6) | 9 (9.4) | 1.67 ± 0.66 |
| Have you ever felt dissatisfied with your body? | 0 (0) | 8 (8.3) | 26 (27.1) | 62 (64.6) | 0.44 ± 0.65 |
| Total score | 11.44 ± 1.99 | | | | |

**Coding: At great deal = 3 , Sometimes = 2 , Rarely = 1 , Never = 0**

**Table S5:** Body Image Scale – post operatively correlation with port site complications after propensity score matching (PSM) (n=96)

|  | **Port site complications** | **No Port site complications** | ***P*-value** |
| --- | --- | --- | --- |
|  | **(n=43)** | **(n=43)** |  |
| **Body Image Scale – Post-operative** | 11.97 ± 1.93 | 11.44 ± 2.23 | 0.290 |

**Fig. S1:** Port map 8 months post-RBSG: (a) veress needle wound, (b) 10-mm umbilical visual port, (c) 2-mm liver retraction wire wound, (d) 12-mm right working port, (e) 15-mm left working port. Image captured prior to laparoscopic cholecystectomy for symptomatic gallstones.

**
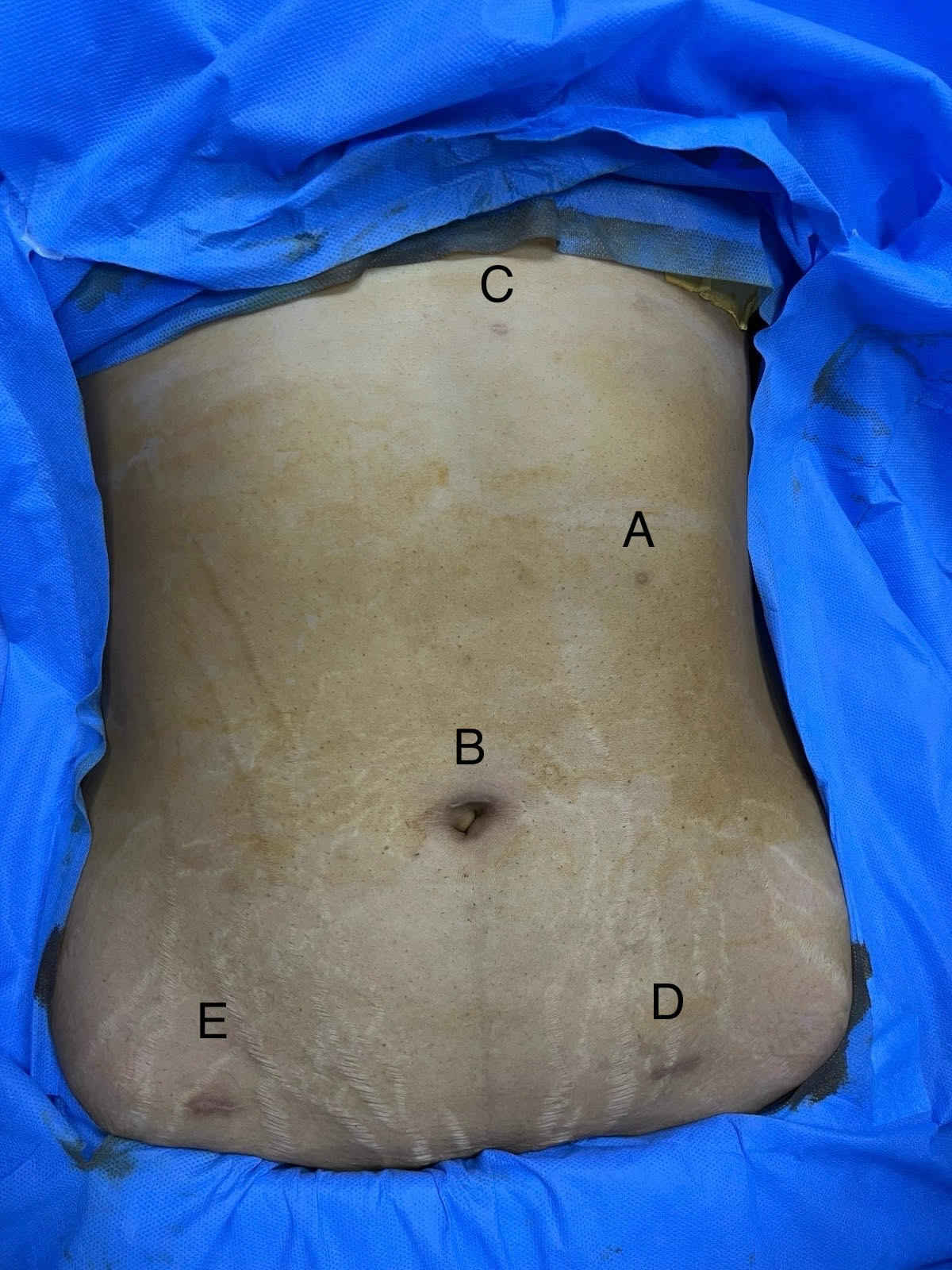
**

**Fig. S2 :** Laparoscopic view of IEA


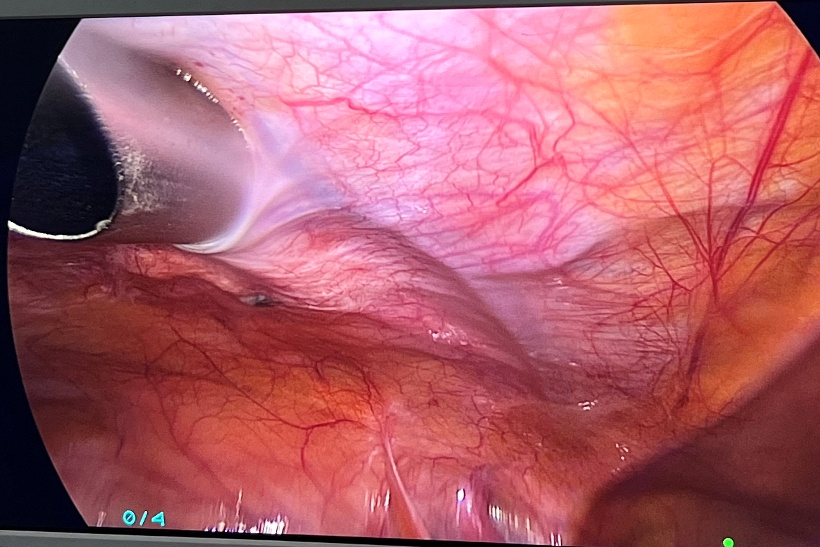


**Fig. S3:** Complication chart


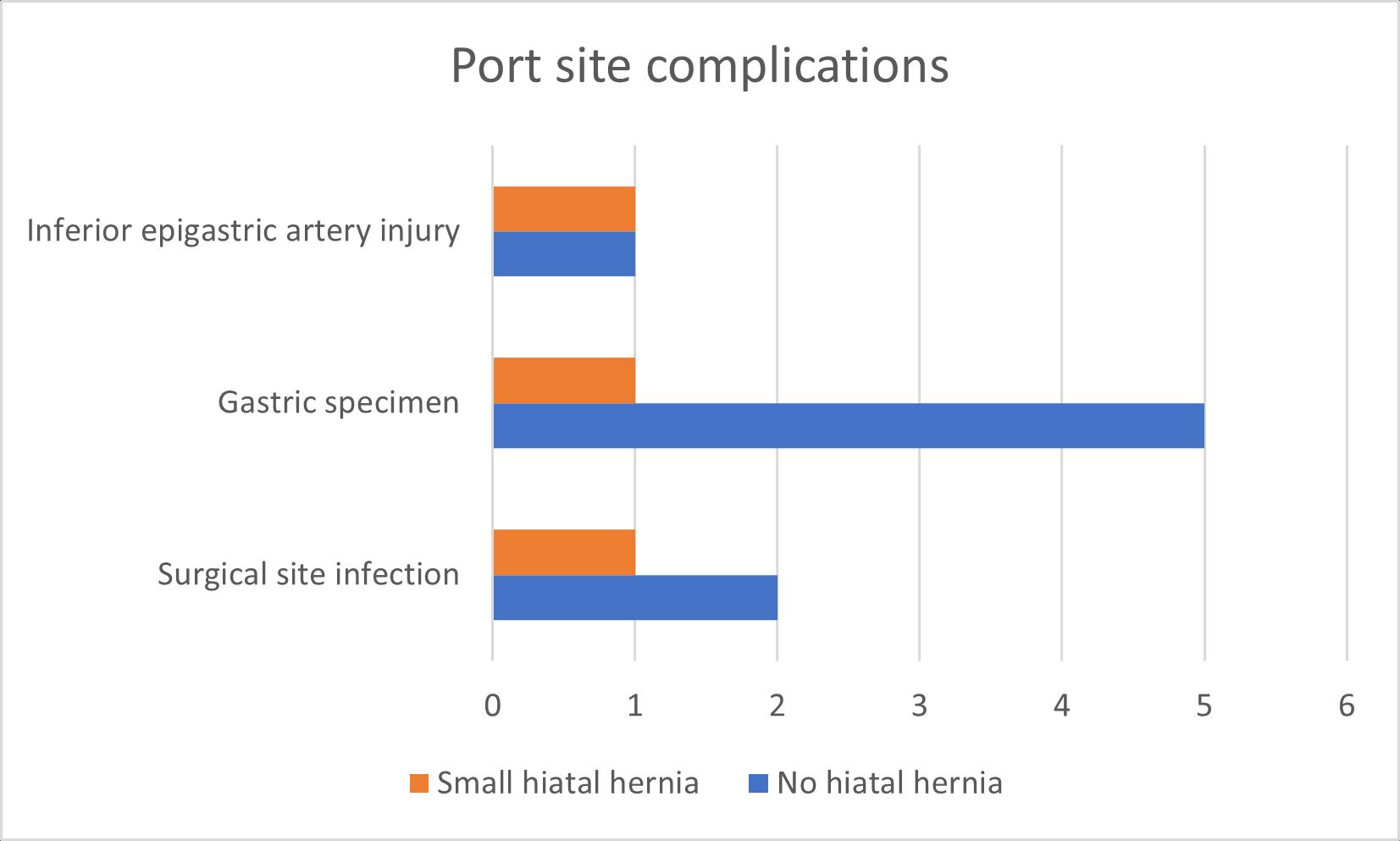


**Fig. S4:** BIS comparison by hernia status(A), BIS vs NRS (pain correlation graph) (B)
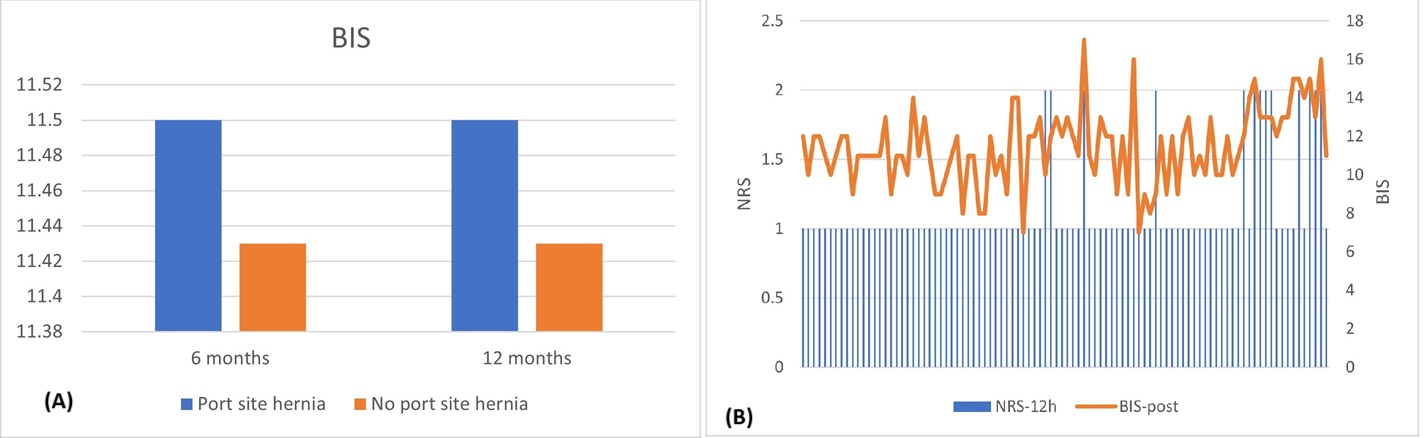

Supplement: Supplementary file 1 — Supplementary file1 (DOCX 650 KB) [file 11695_2025_8176_MOESM1_ESM.docx]
